# Extracting public opinion on typhoon disasters in China: a sina weibo case study of landfalling typhoon Muifa (2022)

**DOI:** 10.1038/s41598-026-40736-8

**Published:** 2026-02-23

**Authors:** Yanran Sun, Qian Wang, Yongchang Zhu, Jing Xu, Lu Liu, Chunyi Xiang, Chuanhai Qian

**Affiliations:** 1 Beijing Information Science & Technology University, Beijing, 102206 China; 2 National Meteorological Centre, China Meteorological Administration, Beijing, 100081 China; 3 Qingdao Institute of Marine Meteorology, Chinese Academy of Meteorological Sciences, China Meteorological Administration, Beijing, 100081 China; 4 CMA Key Open Laboratory of Transforming Climate Resources to Economy, CMA Institute for Development and Programme Design, Beijing, 100081 China

**Keywords:** Typhoon Muifa (2022), Sina Weibo, Microblogs, Social media, Public opinion, Climate change, Environmental social sciences, Natural hazards

## Abstract

This study investigates public opinion dynamics on Sina Weibo during Typhoon Muifa (2022), which made four landfalls in China. Using a dataset of 19,417 microblog posts, we employed Latent Dirichlet Allocation (LDA) topic modeling, sentiment analysis, and correlation statistics to characterize the evolution of public attention and discourse alongside the typhoon’s activity. Results identified four dominant discussion topic categories: typhoon impact, weather conditions, meteorological information, and disaster response. Personal accounts predominantly contributed to the first two categories, while official accounts dominated discussions on the latter two. A strong positive correlation emerged between daily total precipitation and the number of microblog counts (R^2^ = 0.84, q < 0.001), which was particularly pronounced in forecasted landfall provinces Zhejiang, Shanghai, Shandong, and Liaoning (q < 0.05). Negative sentiment was highly correlated with rising precipitation, a trend largely driven by discussions within the typhoon impact topic category. Our findings underscore the potential of social media as a real-time indicator of localized public sentiment during disasters, with official risk narratives playing a key role in shaping attention. This study offers insights that may inform targeted risk communication and emergency management strategies.

## Introduction

Extreme weather events such as typhoons can have profound and extensive impacts on society and the economy^1^, including threats to public safety and potential severe economic losses through strong winds and torrential rains^2^. China, in particular, faces significant challenges due to the severity of typhoon landfalling disasters^3,4^. As typhoons approach, public demand for official forecasts, early warnings, and post-disaster information surges, driving attention to social media platforms, which have become important channels for information access. Prior studies have identified social media’s multifaceted disaster roles: information dissemination, public discourse and emotional expression, and social mobilization^5–7^. Consequently, these platforms have emerged as valuable sources of public opinion data for governments, agencies, and researchers. Therefore, analyzing social media data has emerged as a promising approach to study public discourse during disasters and to potentially inform improvements in disaster management and response. Such analyses typically focus on four dimensions: space, time, content, and network, which aim to enhance situational awareness and understand public discourse^8^.

As a critical information channel, platforms like Twitter enable rapid sharing of official alerts and user-generated updates. Studies show that user anxiety and altruism drive disaster information seeking, monitoring, and redistribution^9^. Hazard-related content spreads faster and wider than reassuring messages, marked by shorter retweet intervals and broader propagation networks^10,11^. Visual content (e.g., statistical graphics, risk maps) also efficiently captures public attention and conveys complex information^12^.

Social media further acts as a public sphere for discourse and emotional expression, where users share concerns, experiences, and navigate disaster-induced emotional stress^13^. Beyond communication, these platforms are highly effective in facilitating social mobilization. The demonstration effect of shared content can inspire collective action^14,15^. This aligns with phased disaster behavioral models during disasters, from initial Awareness, through Preparation, to Action, with social media serving as a pivotal catalyst to translate awareness into preparatory and actionable measures^16^.

The real-time, granular, and voluminous nature of social media data has accelerated its integration into formal emergency management frameworks^17^, offering distinct advantages over traditional sources for disaster detection, early warning, behavioral monitoring, public opinion analysis, and decision support^18–21^. For instance, machine learning-based Twitter data analytics enable rapid damage assessment^22^, while multi-dimensional frameworks integrating geolocation, sentiment, and demographic data support disaster loss prediction and community risk perception assessment^23,24^.

In China, Sina Weibo functions analogously to Twitter^25^, with its emergency management role well-documented in public health crises like the COVID-19 pandemic^26,27^. Regarding natural disasters, studies have utilized Weibo data to analyze public responses to events including floods in Wuhan and Nanjing in 2016, and Super Typhoon Lekima in 2019^28–30^. Collectively, these approaches improve situational awareness and advance understanding of public discourse and response during disasters.

Spatial analysis leverages social media geographic information (e.g., geocoordinates, toponyms) for disaster mapping, risk identification, and damage assessment^31,32^. Wu et al. extracted Sina Weibo victim locations during Super Typhoon Lekima (2019) to develop a multi-source data fusion framework for emergency coordination^30^. Similarly, Wang et al. proposed a Spatial Logistic Growth Model (SLGM) that integrates social media feeds with auxiliary data to characterize the spatial diffusion of citizen-generated disaster awareness data driven by seismic intensity, enabling rapid earthquake-affected zone estimation and validating geosocial data’s utility for situational analysis^33^.

Temporal analysis capitalizes on real-time social media streams for early detection and public response monitoring. Disaster-related awareness can emerge 72 h pre-event, outpacing official channels^34^. Sakaki et al. developed a real-time earthquake detection system via tweet analysis^35^. Spruce et al. documented topic shifts from pre-storm warnings to post-disaster assessments in UK/Ireland winter storm tweets^36^. Multiple studies have confirmed positive correlations between activity levels and disaster metrics, such as severity or meteorological indicators^37,38^.

Content analysis delivers critical decision-making support. Supervised/unsupervised learning such as Latent Dirichlet Allocation (LDA) and word frequency analysis categorizes content to identify dominant themes, including situational updates, opinions, emotions, and other narratives. The evolution of these themes offers a dynamic lens on shifting public concerns, further substantiating the instrumental value of social media for real-time disaster assessment^28^.

Prevalent and detectable emotional patterns make sentiment analysis a key subfield^39^. It analyzes emotional orientations (positive, negative, neutral) to gauge public reaction, and subsequently informs disaster response, psychological interventions, and public opinion management^40^. Negative emotion intensity usually correlates with disaster severity and loss^41,42,43^, which has led to practical applications. Bai and Yu predicted public panic from negative Weibo posts^44^, while Yuan and Liu linked damage-related tweets to Hurricane Matthew (2016) insurance claims^43^. Li et al. created a text-based damage scale and early-stage estimation model using 2019 Ridgecrest earthquake social media data^45^.

Network analysis clarifies information diffusion paths by examining interaction structures. Research shows that information dissemination networks formed during disasters are significantly denser than those during normal periods, a phenomenon characterized by a marked increase in users (nodes) and retweet relationships (edges)^46^. By applying network metrics (e.g., degree, betweenness centrality), researchers can quantitatively detect dissemination patterns and pinpoint pivotal users or communities^47^.

Within this context, this study focuses on typhoons as a specific type of meteorological disaster. It examines the dynamics of public opinion on social media throughout the disaster process and explores how such data can be applied to inform disaster management practices. Typhoon Muifa (2022) was selected for its unprecedented four landfalls in China, as well as its substantial ensemble track spread and pronounced landfall uncertainty in forecast. Its unique trajectory offered a valuable case to observe how public opinion evolves in relation to the sequential release of forecasts and warnings. This case study was designed to address the identified research gaps regarding opinion dynamics during sequential hazard events.

## Data and methods

### Data collection

All times referenced in this study are in Beijing Time (UTC + 8). Subsequent data collection and analysis are conducted based on this time zone.

The track, maximum sustained wind speed (Vmax), and minimum sea level pressure (SLP) of Muifa (2022) were obtained from the best-track dataset^48,49^ that was analyzed by Shanghai Typhoon Institute of the China Meteorological Administration (STI/CMA) at 6-h intervals (http://tcdata.typhoon.org.cn). The hourly TC positions were obtained from the official typhoon bulletins issued by the National Meteorological Center of the China Meteorological Administration (NMC/CMA). When a typhoon approaches China, its position and intensity information are released every 3 h within the 48-h warning zone and are updated hourly within the 24-h warning zone. Typhoon early warnings are issued by CMA three times a day, at around 06:00, 10:00 and 18:00 respectively. Precipitation of Muifa (2022) from 08:00 September 11 to 08:00 September 18, 2022 were acquired from the National Weather Information Center of CMA (http://data.cma.cn), including station name, latitude, longitude and 1-h precipitation value.

For public opinion analysis, Sina Weibo microblogs served as the data source. The data were collected using the Sina Weibo API, which is provided to the NMC for academic research. The API’s built-in filtering mechanisms were utilized during the initial data retrieval to minimize content from non-human or bot accounts. We searched the Sina Weibo archive for posts containing both the keyword “Typhoon Muifa” and the hashtag “#Typhoon Muifa#”, covering the period from September 6 to 20, 2022. Following data retrieval, our data partner conducted a secondary technical screening in an effort to further identify and remove posts suspected to originate from “zombie” accounts or bot activity. This process yielded two preliminary datasets: one containing 20,130 posts mentioning “Typhoon Muifa” and another containing 7,126 posts with the hashtag “#Typhoon Muifa#”. After merging the two datasets, we performed data cleaning by removing duplicates, filtering out identical contents and URLs, and converting emojis into text.

### Topic modeling and evaluation

Topic modeling is one of the most powerful techniques in text mining. Among these, Latent Dirichlet Allocation (LDA), initially introduced by Blei et al.^50^, is one of the most widely used methods^51^. As a generative probabilistic model for a corpus, its fundamental concept is that documents are depicted as random combinations of underlying topics, with each topic being distinguished by a distribution of words^50^.

In this study, we first performed tokenization using the jieba.lcut(text) function from the jieba library in Python. Jieba is particularly suited for Chinese text segmentation as it effectively splits text into individual words or tokens. After tokenization, stopwords were removed. Stopwords are common words that do not carry significant meaning in analytical contexts, such as interjections, conjunctions, and prepositions, for example, “啊” (ah), “和” (and), “能够” (can), “对于” (regarding).

The cleaned texts were then transformed into a document-term matrix. In this matrix, each row corresponds to a microblog post, each column represents a unique word, and each element indicates the frequency of a specific word in the corresponding post. The LDA model iteratively optimizes this matrix to construct a topic model, thereby calculating the probability distribution of each word across the different topics.

**Fig. 1:**
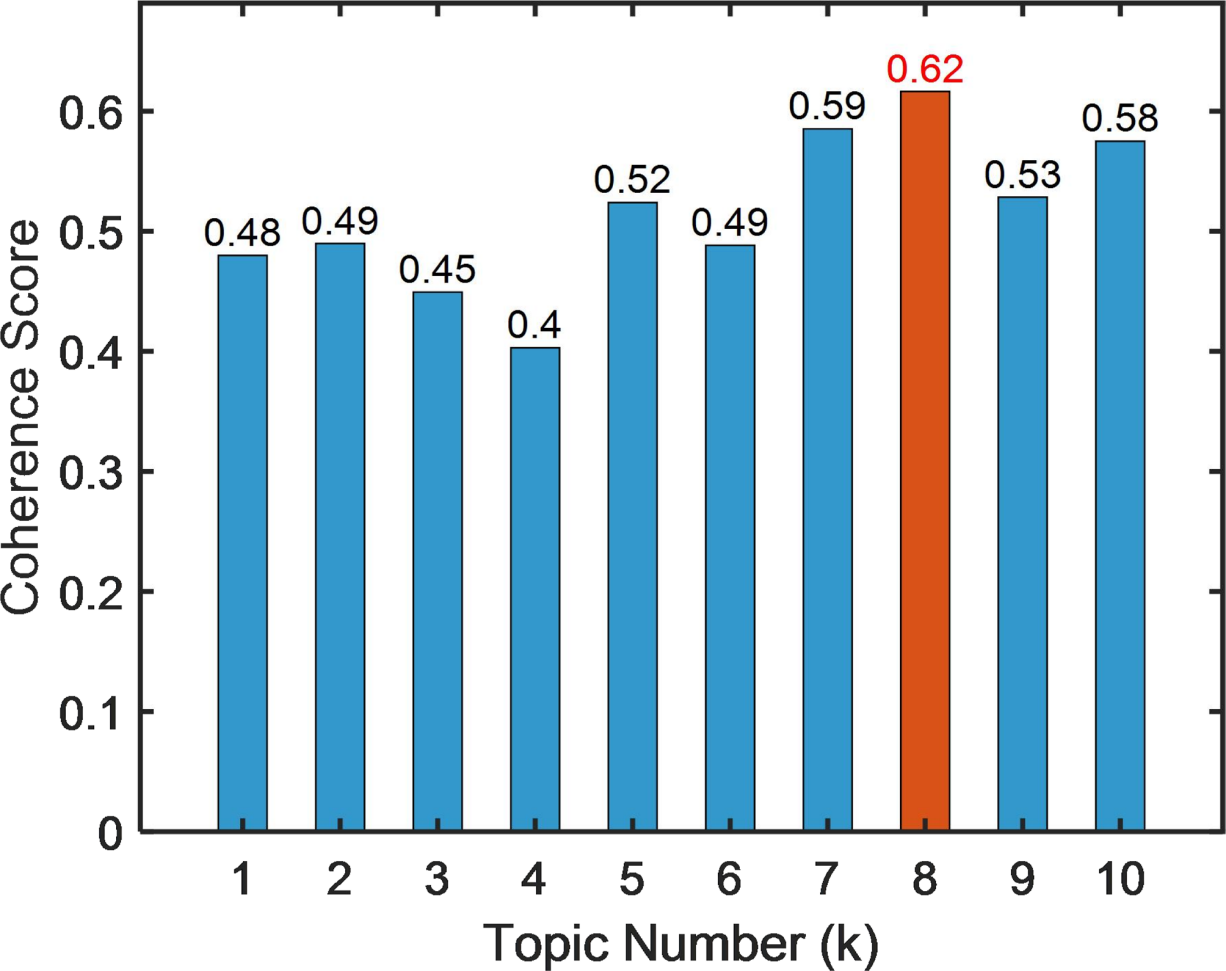
Bar chart of topic coherence scores for determining the optimal number of topics. The highest score at k = 8 identifies it as the optimal number of topics for the analyzed corpus, providing the most semantically coherent topic model.

To determine the optimal number of topics, we employed the CoherenceModel module from the gensim library, which uses the Cv coherence measure proposed by Röder, M et al^52^. The coherence value, defined as the average of semantic similarity scores among words within a topic, measures the internal consistency of topics and reflects the quality of topic segmentation. A higher coherence value indicates that the words within a topic are more semantically related, implying a more interpretable topic structure^53^. We evaluated topic numbers ranging from 1 to 10. A topic number of 8 was selected, based on the trend of coherence scores (Fig. 1), where the coherence score reached a peak. This suggests that, within the tested range of 1 to 10 topics, the model with 8 topics produced the most semantically coherent topics for our corpus.

### Theme categorization and validation

The LDA model generated 8 topics based on word co-occurrence patterns. To facilitate a higher-level semantic analysis, we manually merged these 8 data-driven topics into 4 broader, more interpretable macro-categories. This merging process was guided by their semantic coherence and was theoretically informed by the disaster management lifecycle framework, which is often conceptualized in stages such as preparation, response, and recovery^54^. We hypothesized that public discourse on social media during a typhoon event might broadly align with phases of the disaster management lifecycle. For example, discussions focusing on “warning information” corresponded to the preparation stage, while those concerning “school suspension” or “resumption of work” reflected the impact and recovery stages, respectively. This conceptual framework guided our manual integration of the topics into the macro-categories: typhoon impact, weather conditions, meteorological information, and disaster response. Detailed definitions and the specific merging scheme are provided in Sect. 3.3.1 and Table 1.

To ensure the validity and reliability of this categorization, we conducted a systematic manual validation. First, a validation set of 400 microblog posts was constructed. This set was created by randomly sampling the 50 most representative posts from each of the 8 original topics, a procedure which applied weighting based on topic probability. Subsequently, two coders familiar with the research context were trained using the definitions and examples of the four macro-categories. They then independently assigned each post in the validation set to one of these categories. Finally, we assessed the inter-rater reliability of their assignments by calculating Cohen’s Kappa coefficient.

### Sentiment analysis

We employed the pre-trained model “roberta_chinese_large” (https://huggingface.co/clue/roberta_chinese_large) for sentiment analysis. This model is based on the RoBERTa (Robustly Optimized BERT Approach) architecture, a variant of the BERT (Bidirectional Encoder Representations from Transformers) model. RoBERTa is a transformer-based architecture designed for natural language processing (NLP) tasks that achieves enhanced performance by modifying training objectives and data generation methods, as well as by increasing training scale^55^. The specific “roberta_chinese_large” model was pre-trained on the CLUECorpusSmall dataset (https://github.com/CLUEbenchmark/CLUECorpus2020).

We fine-tuned this model for our specific task, which involved further training it on labeled microblog data while preserving most of its pre-trained weights. The fine-tuning was conducted for 20 epochs with a dropout of 0.1, a learning rate of 1e-6, and a batch size of 48. The model converged at epoch 5 with an accuracy of 0.793, and an F1 score of 0.765 on the validation set. Then we tested the model on the test set, obtaining F1 and accuracy values of 0.766 and 0.791, indicating that the model is viable.

The fine-tuned RoBERTa model was subsequently used to predict the sentiment of each microblog post.

**Fig. 2:**
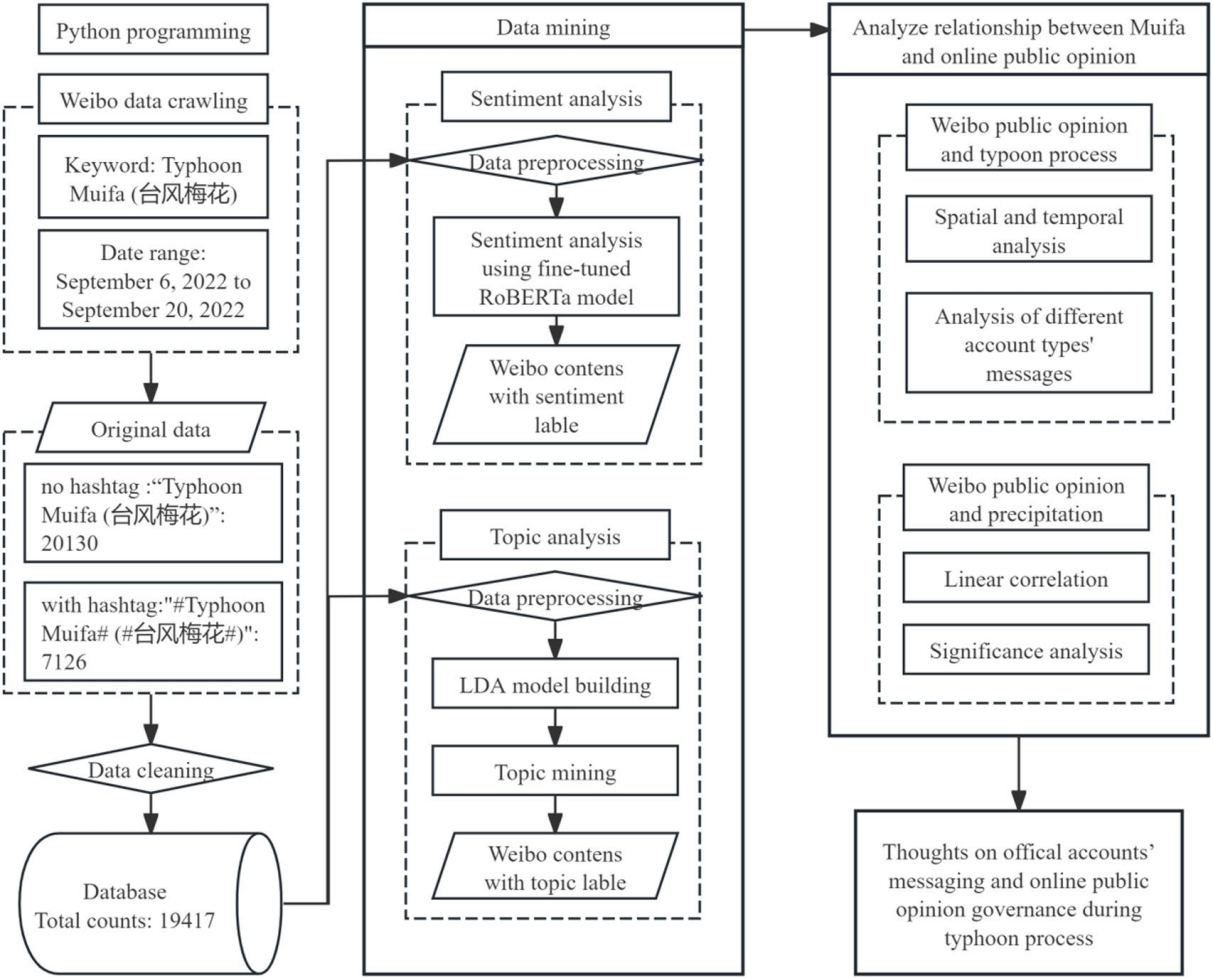
Technical flow chart of this study.

The complete technical workflow encompassing data collection, topic modeling, evaluation, and sentiment analysis is summarized in Fig. 2.

## Results

### A brief history of Typhoon Muifa (2022)

**Fig. 3:**
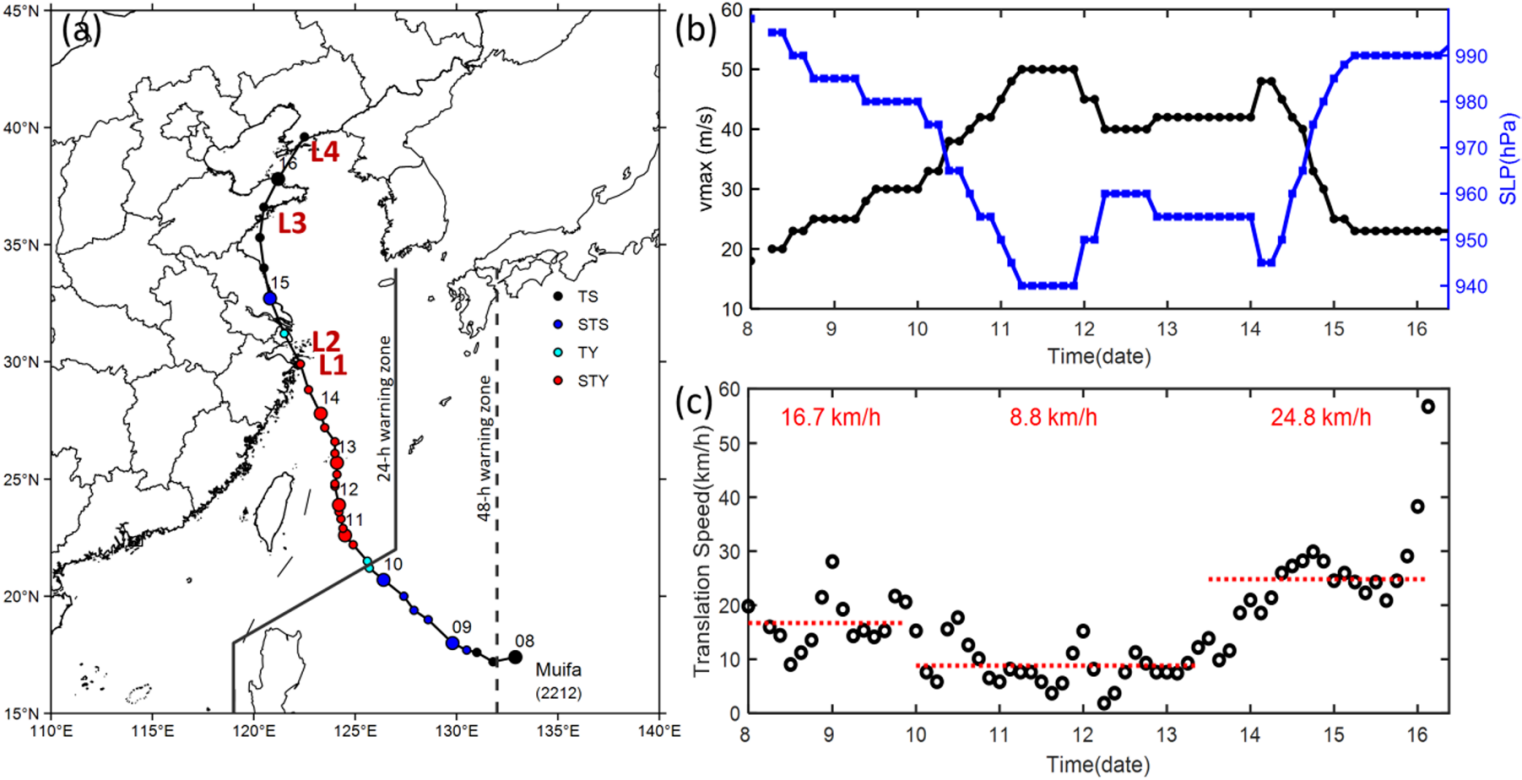
Track, intensity, and translation speed of Typhoon Muifa (2022). (**a**) Track of Muifa (2022) in 6-h intervals. The tropical cyclone (TC) intensity categories follow the Grade of Tropical Cyclones (GB/T 19201 − 2006), including tropical storm (TS), severe tropical storm (STS), typhoon (TY), and severe typhoon (STY). Numbers adjacent to the symbols denote the dates in September 2022. Four landfalls illustrated subsequently as L1, L2, L3 and L4. (**b**) Time series of the maximum sustained wind speed (unit: m s^−^^1^) and minimum sea level pressure (SLP) (unit: hPa). (**c**) Time series of translation speed (unit: km h^−^^1^) of Typhoon Muifa (2022) from 08:00 on September 8 to 17:00 on September 16, 2022.

Typhoon Muifa (2022) was a significant typhoon that affected China in mid-September 2022^56^, notable for making four landfalls. The dissemination of warning information plays an important role in disaster risk reduction via the early warning value chain assessment^57^. Muifa (2022) formed over the warm ocean east of the Philippines at 08:00 on September 8. Thereafter, it moved northwestward, intensifying into a typhoon by 08:00 on September 10 and further to a severe typhoon 12 h later (Fig. 3a). After turning northward and moving slowly for two days, during which it underwent cycles of weakening and re-intensification, the typhoon bypassed the sea east of Taiwan Island and entered the East China Sea (ECS). On 13 September, Muifa (2022) moved north-northwestward toward Zhejiang Province. It intensified offshore on the morning of September 14 (Fig. 3b), and made its first landfall around 20:30 near Zhoushan, Zhejiang as a severe typhoon (42 m s^−^^1^). After crossing the Hangzhou Bay, it made second landfall at 00:30 on September 15 in Shanghai as a typhoon (35 m s^−^^1^). It continued moving northward and made its third landfall as a tropical storm (23 m s^−^^1^) in Qingdao, Shandong at approximately 00:00 on September 16. The typhoon then turned northeastward, making its fourth and final landfall in Dalian, Liaoning (23 m s^−^^1^), at 11:25 on the same day.

Typhoon Muifa (2022) was the first typhoon on record to land in four different provinces/municipalities and was the strongest landfalling typhoon in 2022. Its prolonged track caused extensive wind and rain impacts across the East and Northeast China. The forecast track presented considerable uncertainty, partly due to the influence of multiple synoptic-scale vortices on its movement. This uncertainty generated significant public discussion regarding the track forecast on Sina Weibo. As Muifa approached Zhejiang, its translational speed was slow, averaging approximately 8.8 km h^− 1^ from 08:00 on September 10 to 20:00 on September 13 (Fig. 3c). Its trajectory also shifted northward around September 12, a trend that initially suggested a lower probability of landfall in China, biasing the original forecast track and resulting in an inaccurate landfall forecast. However, the movement subsequently shifted toward the northwest, which ultimately led to landfall in Zhejiang and facilitated accurate track prediction and decision-making.

### Evolution of public opinion on Sina Weibo

Typhoon Muifa (2022) entered the 48-hour warning zone after 14:00 on September 8 and the 24-hour warning zone after 14:00 on September 10. In accordance with forecasting service requirements, the NMC began issuing a typhoon blue warning at 06:00 on September 11. This action coincided with the increase in official information updates from every three hours to hourly after the typhoon entered the 24-hour warning zone. A concurrent surge in Sina Weibo user activity was observed. The warning level was subsequently upgraded to yellow at 18:00 on September 12, to orange at 06:00 on September 13, and finally reached the highest red alert at 10:00 on September 14. During this period, the official NMC account published over 160 microblog posts, which accumulated more than 35 million views.

**Fig. 4:**
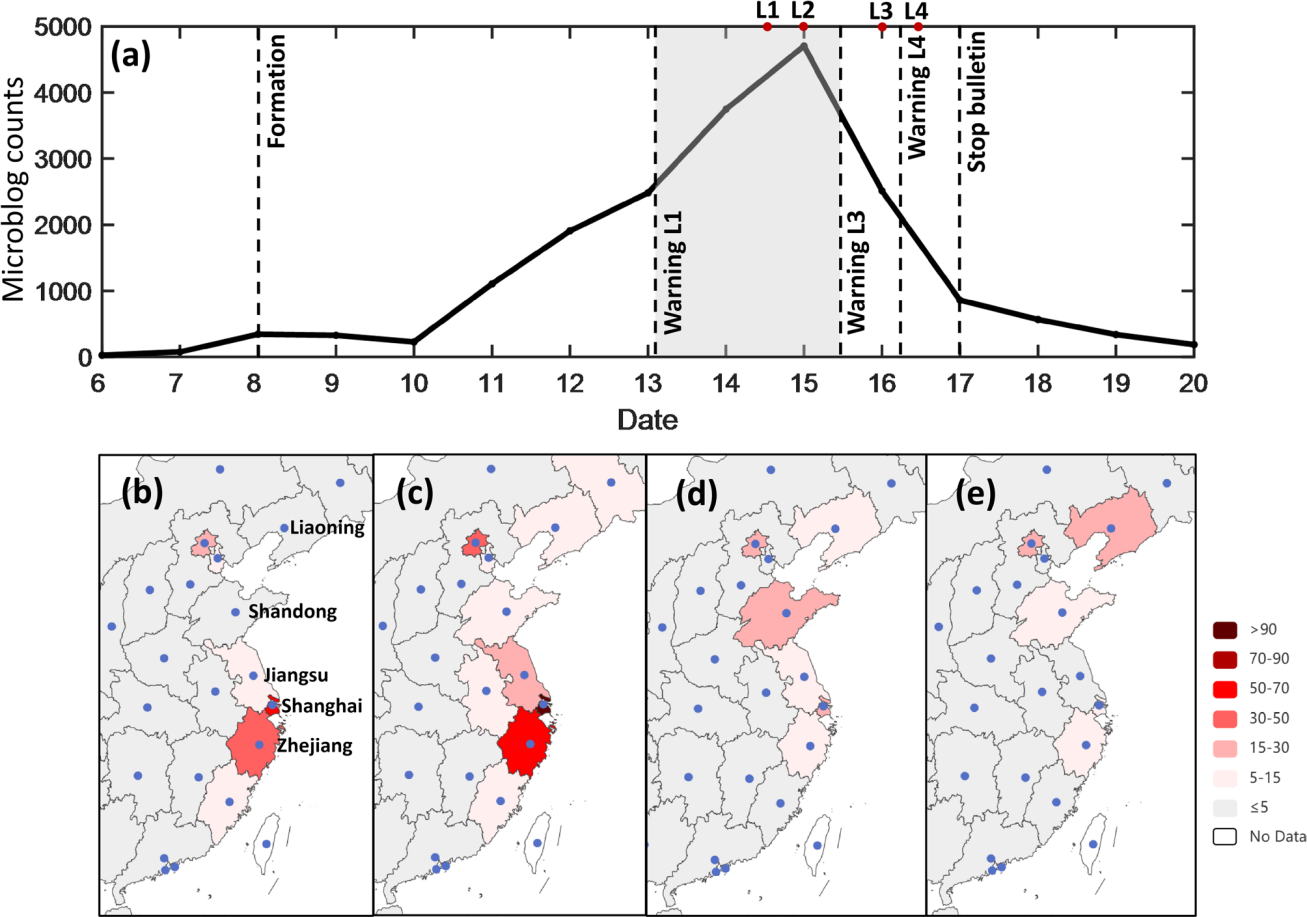
Temporal trend and spatiotemporal evolution of public attention on Sina Weibo during Typhoon Muifa (2022). (**a**) Daily count of microblog posts related to Typhoon Muifa from September 6 to 20, 2022. (**b-e**) Geographic distribution of the provincial-level Standardized Weibo Attention Index (calculated from Eq. 1) across four key stages of the typhoon’s lifecycle: (**b**) the formation and early warning stage (from 10:03 on September 8 to 08:49 on September 13), (**c**) the first and second landfall stage (to 09:03 on September 15), (**d**) the third landfall stage (to 09:06 on September 16), and (**e**) the fourth landfall and dissipation stage (to 08:21 on September 17).

This study analyzed microblog data related to Typhoon Muifa from September 6 to 20, 2022. The daily post volume varied significantly over this 15-day period (Fig. 4a). The mean daily post count was 1,234, while the median was 568, indicating that discussion volumes were generally moderate on most days but experienced extreme spikes on a few occasions. The data showed considerable dispersion, with a standard deviation (SD) of 1,440 and a range of 4,678. This dispersion corresponded to a substantial increase in public attention as the typhoon approached and made landfall between September 12 and 16. The daily post count peaked at 4,704 on September 15, when the typhoon was at its strongest intensity. This peak was 180 times higher than the lowest daily count of 26, recorded on September 6. The observed pattern reflects a “concentrated outbreak” model, where public discussion is dominated by episodic bursts of attention that coincide with the peak of a meteorological crisis.

We further characterized the dataset by examining the distribution of microblog post lengths and user activity patterns. The distribution of post length was notably bimodal. The majority of posts (62.95%) contained 140 characters or fewer, with a noticeable concentration (19.51%) in the 21–40 character range; these typically represented brief comments or shared headlines. Meanwhile, 37.05% of posts exceeded 140 characters, which suggests that a substantial portion of users engaged in more detailed descriptions or multi-point discussions about the typhoon. This dichotomy reflects a communication spectrum ranging from concise, real-time updates to more elaborate, narrative-style posts.

User activity patterns revealed a highly skewed distribution. The vast majority of users (75.37%) posted only once about the typhoon, indicating widespread but transient engagement from the general public. In contrast, a very small core group of highly active users (0.07%) posted more than 50 times each. This group likely included weather enthusiasts, emergency responders, journalists, or influencers, who played a disproportionate role in sustaining and shaping the online conversation. This pattern underscores a typical structure in crisis communication on social media: a large, diffuse audience contributes intermittently, while a narrow, highly vocal cohort drives continuous information flow and discussion.

To examine the temporal and regional trends in microblog activity across different stages of Muifa, this study used typhoon forecast information posted by the “@NMC” account (the official account of China’s National Weather Forecasting Center) as a reference framework. We quantified the number of microblog posts from various regions across four defined stages, with the geographical origin of posts determined through platform-derived user profile data.

The first stage covered the typhoon formation period (Fig. 4b). It began at 10:03:21 on September 8, 2022, when @NMC posted an announcement regarding the formation of Typhoon Muifa: “#Typhoon# Typhoon Muifa (TS) (name from Macau, China; meaning: a type of flower), the twelfth typhoon of this year, has formed. It is expected to reach strong typhoon or super typhoon at its peak intensity. Currently, it is forecasted to enter the ECS and move northward on the 12^th^. The affected areas include the ECS and the coastal regions of East China. Stay tuned for updates!”

The second stage encompassed the landfalls in Zhejiang and Shanghai (Fig. 4c). It was defined from 08:49:45 on September 13, when @NMC posted that the typhoon would make landfall in Zhejiang: “Typhoon ‘Muifa’ will make landfall in Zhejiang tomorrow. Orange typhoon warning issued.” Due to the short time interval and geographical proximity of the first two landfalls, their statistics were combined for clarity.

The third stage corresponded to the typhoon’s third landfall in Shandong (Fig. 4d). This stage began at 09:03:44 on September 15, marked by an @NMC post forecasting the third landfall: “Typhoon ‘Muifa’ may make its third landfall in Shandong tonight. Shandong, Liaoning, and Jilin will experience continuous heavy rainfall.”

The fourth stage spanned from the fourth landfall in Liaoning to the typhoon’s dissipation (Fig. 4e). It began at 09:06:40 on September 16 with an @NMC post about the impending fourth landfall: “Typhoon ‘Muifa’ will make its fourth landfall in China, potentially breaking the record for the latest typhoon landfall in Liaoning.” And the stage ended at 08:21:21 on September 17 as @NMC posted: “Typhoon ‘Muifa’ has transformed into an extratropical cyclone in northeastern Liaoning and was finally reported at 20:00 last night. …”.

Data analysis traced the evolution of public attention as Typhoon Muifa moved northward. During the typhoon’s formation and initial approach, eastern coastal provinces like Zhejiang and Shanghai dominated the online discussion. This focus culminated during the first and second landfalls, with Zhejiang, Shanghai, and Jiangsu generating 2,428, 1,093, and 873 posts respectively. However, the absolute volume of posts gradually decreased in subsequent stages (Fig. 4a) as the typhoon weakened.

To enable a comparative assessment of public attention levels across provinces with differing internet user bases, we standardized the microblog volume using provincial internet broadband subscriber data^58^. The Standardized Attention Index for province $\:p\left({I}_{p}\right)$ using the following Eq. (1):

1 $$\:{I}_{p}=\frac{{W}_{p}}{{S}_{p}}\times\:1000$$

where $\:{W}_{p}$ is the total number of relevant Weibo posts from province $\:p$, and $\:{S}_{p}$ is the number of broadband access subscribers in province $\:p$ (in units of thousand households).

The standardized results (Fig. 4b-e) reveal a consistent pattern of localized attention spikes that raw counts obscure. For example, during the third landfall stage, Shandong’s Standardized Attention Index (17.1) rose to become the highest among affected provinces (Fig. 4d), signaling a peak in relative public concern there. Similarly, during the fourth landfall, the index for Liaoning peaked at 20.2, clearly marking the shift in localized attention despite lower absolute post numbers (Fig. 4e).

The evolution of this index demonstrates that while the overall discussion volume decreased after the first landfalls (Fig. 4a), the relative attention within the immediately threatened area peaked distinctly during each successive landfall event. This pattern indicates that social media activity, when normalized for user base, can precisely mirror the shifting geographical footprint of a disaster’s localized impact.

### Topic analysis

#### Topic analysis with the LDA model

We saved the dictionary and model files of the LDA topic model (with 8 topics) that achieved the highest coherence value during training. Subsequently, these files were loaded using a Python script to extract the top 10 keywords with the highest weights for each topic and to predict the topic of each microblog post.

The eight data-driven topics were manually merged into four macro-categories based on their semantic coherence (Table 1): typhoon impact (Topics 1 and 5), described how Muifa affects socio-economic operations, including immediate disruptions (e.g., traffic suspensions, business closures, school suspensions) and subsequent recovery processes (e.g., work resumption); weather conditions (Topic 2) captured descriptions of real-time atmospheric phenomena (e.g., rainbows, sunsets) shared by the public; meteorological information (Topics 3 and 4) contained official forecasts, early warnings, and physical parameters of the typhoon (e.g., wind speed, central pressure, landfall estimation); and disaster response (Topics 6, 7, and 8) depicted government-led emergency responses and rescue operations.

To validate the subjective rationality of this categorization, we conducted a manual coding validation (see Sect. 2.3). The inter-rater reliability, measured by Cohen’s Kappa coefficient, was 0.827, which indicates an “almost perfect” level of agreement^59^. These four macro-categories were used for subsequent spatiotemporal and sentiment analysis.

**Fig. 5:**
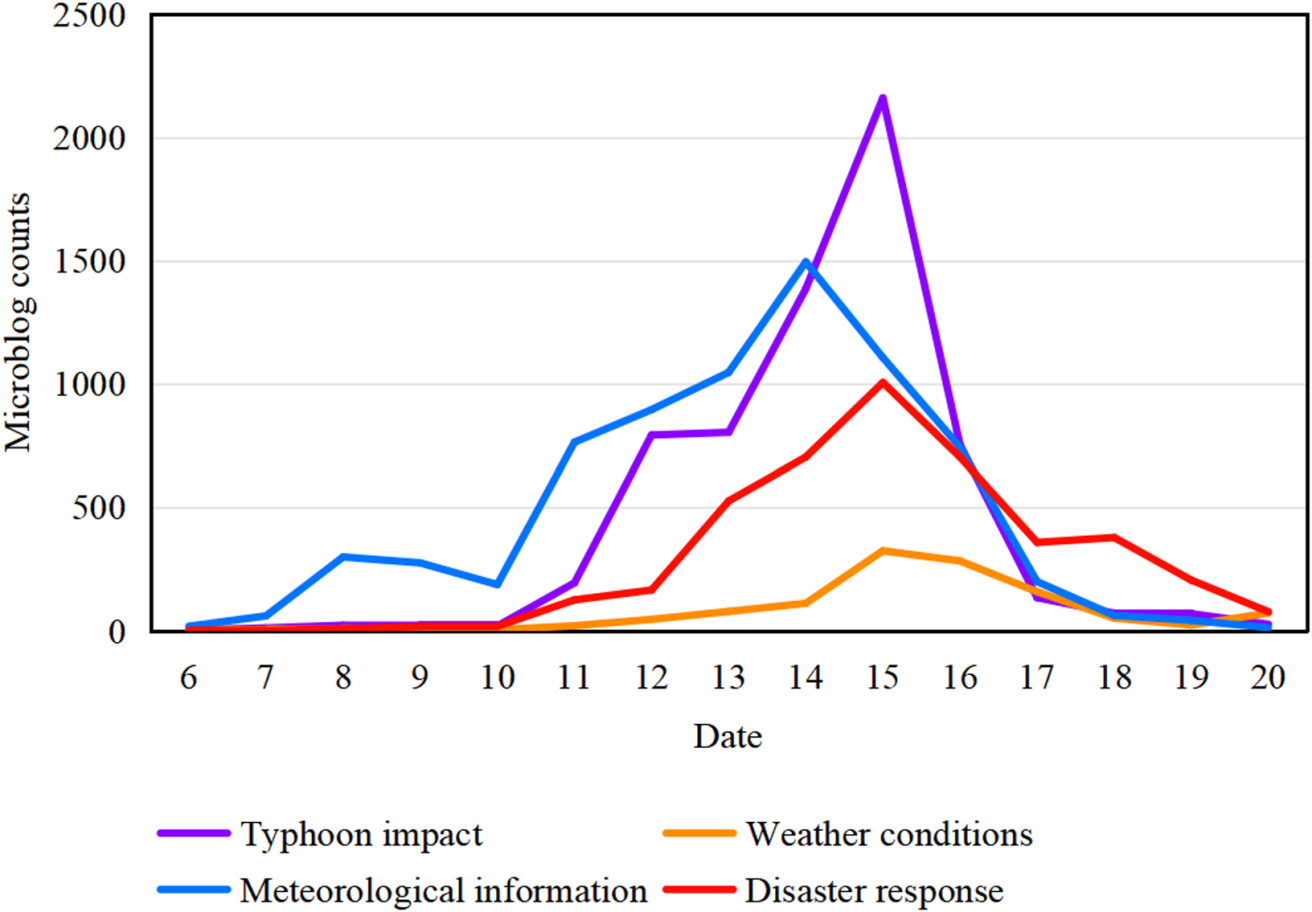
Daily microblog counts across the four topic categories from September 6 to 20, 2022, as derived from the LDA topic model. The plots for the following categories are shown as typhoon impact (purple), weather conditions (orange), meteorological information (blue), and disaster response (red).

Among the 19,417 microblog posts analyzed, 267 posts were assigned to a “no topic” category. This occurred because the model assigned a near-zero probability across all eight topics to these posts, likely due to very short content or a lack of substantive vocabulary (e.g., containing only symbols or function words). It is important to note that posts from this “no topic” category were excluded from all subsequent topic-related statistical analyses and visualizations (i.e., Figs. 5, 7 and 8, and 9c). This category is also not listed among the thematic groups defined in Table 1.

**Table 1:** LDA topic model for microblog content.

Topic Category	Topic No.	Top 10 Words and Probability (in parentheses) from LDA Model
**Typhoon impact**	**1**	Impact (0.014), Shanghai (0.009), Weather (0.008), Suspension (0.008), Flights (0.006), Ningbo (0.006), Coming (0.006), Resumption (0.006), Airport (0.006), Temporary (0.006)
	**5**	School suspension (0.017), Shanghai (0.007), Work (0.006), Elderly (0.005), Rain (0.005), Ningbo (0.005), Water (0.004), School (0.004), Impact (0.004), Holiday (0.003)
**Weather conditions**	**2**	Sky (0.008), Zhejiang (0.006), Sunset (0.005), Hangzhou (0.005), Far away (0.004), Feel (0.004), Rainbow (0.004), Gorgeous (0.004), Present (0.004), Beautiful (0.003)
**Meteorological information**	**3**	Center (0.092), Wind (0.033), Severe typhoon (0.028), Estimated (0.023), Pressure (0.020), Speed (0.020), Sea surface (0.016), Minimum (0.016), Zhejiang Province (0.015), Intensity (0.014)
	**4**	Landfall (0.039), Impact (0.027), Coastal (0.021), Zhejiang (0.020), Warning (0.017), Estimated (0.016), Weather (0.013), Shanghai (0.012), Severe typhoon (0.011), Zhoushan (0.011)
**Disaster response**	**6**	Emergency (0.021), Response (0.015), Impact (0.010), Start (0.009), Work (0.009), Ningbo (0.008), Zhejiang (0.007), Flood prevention (0.007), Defense (0.007), Flood prevention and typhoon response (0.006)
	**7**	Zhejiang (0.004), Rescue (0.004), Transfer (0.003), Elderly (0.003), Ningbo (0.003), Stagnant water (0.003), Trapped (0.003), Resettled (0.002), Auxiliary Police (0.002), Coming (0.002)
	**8**	Trapped (0.012), Shandong (0.012), Yantai (0.010), Waves (0.010), Citizens (0.009), Evacuation (0.009), Personnel (0.008), Impact (0.008), Sea foraging (0.007), Ships (0.007)

#### Topic temporal trend

We analyzed the temporal trends of the four topic categories from September 6 to 20, 2022. As shown in Fig. 5, “meteorological information” was the most prominent topic from September 6 to 11. Beginning on September 12, the topic “typhoon impact” surged in popularity, peaked on September 15, and then declined rapidly. The topics “meteorological information” and “disaster response” also increased in attention starting September 12, although their peak popularity remained significantly lower than that of “typhoon impact”. Notably, the popularity of “meteorological information” declined swiftly and had largely dissipated after September 17. In contrast, attention to “disaster response” persisted longer, with notable activity still present from September 17 to 19.

### Sentiment analysis

#### Sentiment evolution

Using the fine-tuned RoBERTa model, we categorized the sentiment of microblog content into three types: positive, negative, and neutral. The classification results for the full dataset are summarized in Table 2.

**Table 2:** Examples and counts of microblog posts in each sentiment category.

Category	Examples	Counts
Positive	“[#Mom and Dad’s New Blue Sky and White Cloud Profile Pictures# Cotton-like clouds fill the sky of Nanjing after the Typhoon] On September 16th, after Typhoon “Muifa” passed, the weather in Nanjing, Jiangsu Province was sunny and clear, with fluffy “cartoon-like clouds” floating in the sky, making people feel as if they were in a fairy tale world, embracing romance. Netizens: Mom and Dad’s new blue sky and white cloud profile pictures are here!”	4215
Negative	“My mom asked me to go downstairs and throw out the trash. It wasn’t raining when I went down, but after I threw out the trash, it started pouring like there was a leak in the sky [Bye][Bye][Bye]. It looks like the dangerous semi-circle has started hitting Shanghai. This Typhoon Muifa doesn’t blow during work hours, but it starts blowing fiercely when I’m off work, and it keeps blowing all night. Then, when morning comes, it stops blowing. Tomorrow morning, the winds will be calm and the waters still, but you still have to go to work. It shouldn’t be called Muifa, it should be called “Capitalist” [Bye]. #TyphoonMuifa#”	3765
Neutral	“#Typhoon Muifa# [High Incidence of Typhoon and Heavy Rain: What to Pay Attention to for Driving Safety?] If you encounter water accumulation while driving, do not blindly attempt to cross it. If your vehicle gets stuck and stalls in the water, you should quickly open the windows, doors, and sunroof to escape to a safe area. If they cannot be opened, use sharp objects to break the four corners of the window glass. For more safety knowledge, watch the video below (via: Ministry of Emergency Management)”	11,437

**Fig. 6:**
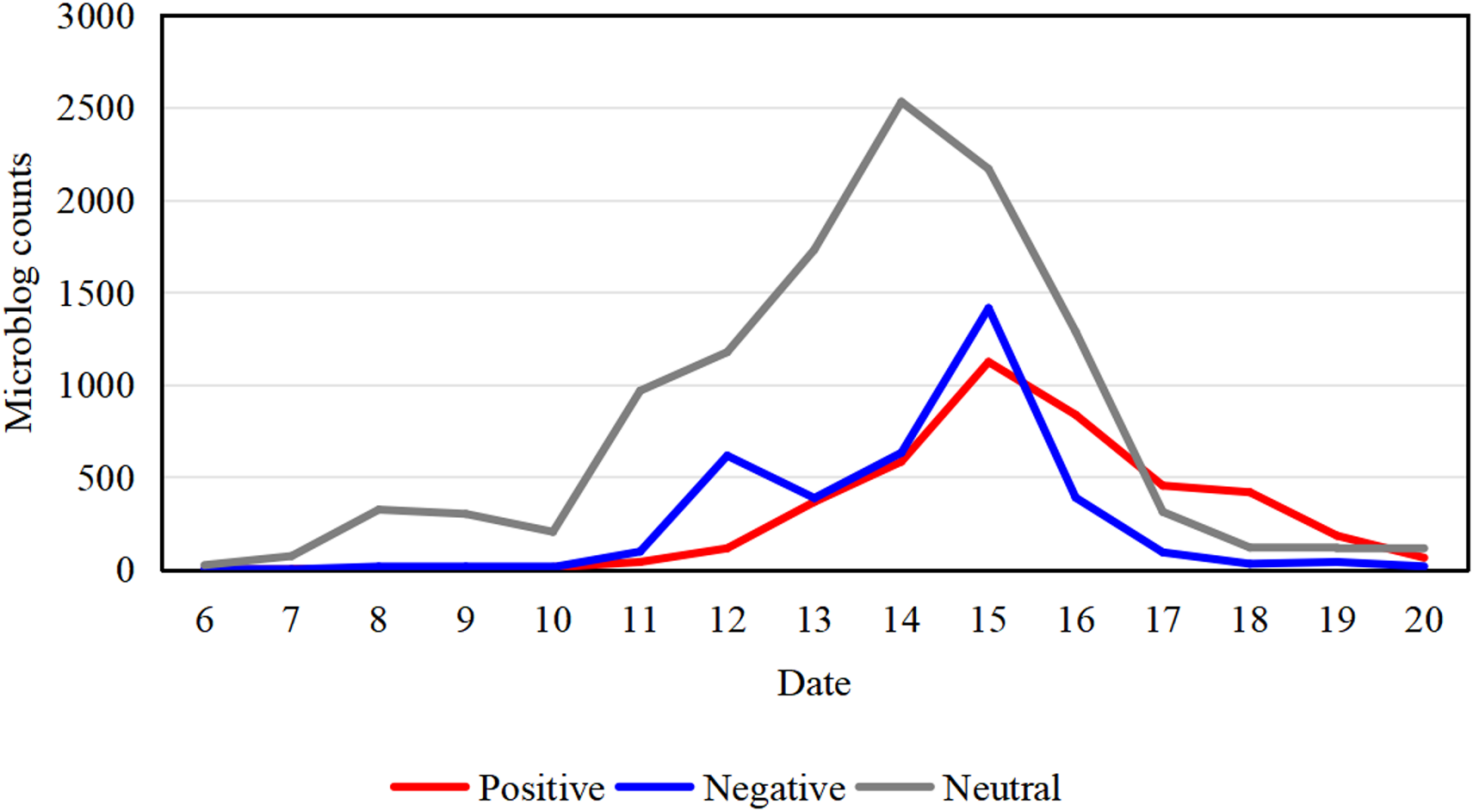
Daily microblog counts classified by sentiment. Sentiment is denoted as positive (red), negative (blue), and neutral (gray). Statistical analysis from 00:00 on September 6 to 23:59 on September 20, 2022.

During the typhoon period, neutral sentiment accounted for the largest proportion of content, and its daily volume trend closely followed the overall trend in total microblog posts. Negative sentiment reached two relative peaks on September 12 and 15 but subsided quickly after each. Positive content was sparse before September 12, gradually increased thereafter, peaked on the 15th, and subsequently subsided at a slower rate than negative sentiment (Fig. 6).

By correlating these trends with the thematic analysis (Fig. 8), we found that the sentiment peaks can be interpreted within the context of the typhoon’s progression. Negative sentiment was primarily associated with the “typhoon impact” topic, which explains its peaks during periods of severe weather. Positive sentiment, in contrast, was largely composed of news and updates related to “disaster response”. Its increase therefore followed the onset of damage and the initiation of relief efforts, exhibiting a lag relative to the impact stage.

Furthermore, we noted that the collective memory of the 2011 typhoon of the same name (Muifa), which famously deviated from forecasted paths and avoided landfall, was actively discussed online prior to September 12. This historical context may have influenced public sentiment during the 2022 event by shaping perceptions of forecast reliability, which could explain the intensity of negative reactions observed on September 12.

#### Sentiment distribution across topics

**Fig. 7:**
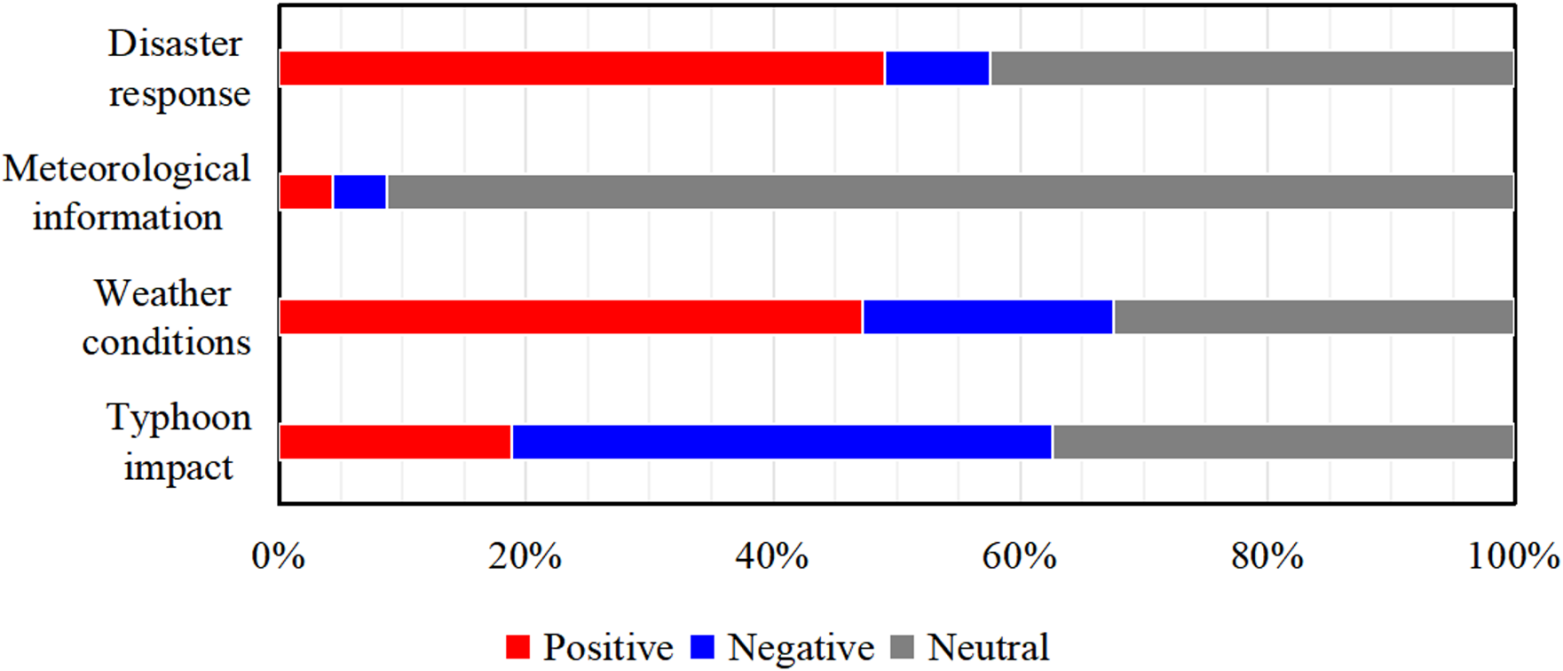
Proportional distribution of sentiment across the four topic categories. Sentiment is denoted as positive (red), negative (blue), and neutral (gray). Statistical analysis from 00:00 on September 6 to 23:59 on September 20, 2022.

**Fig. 8:**
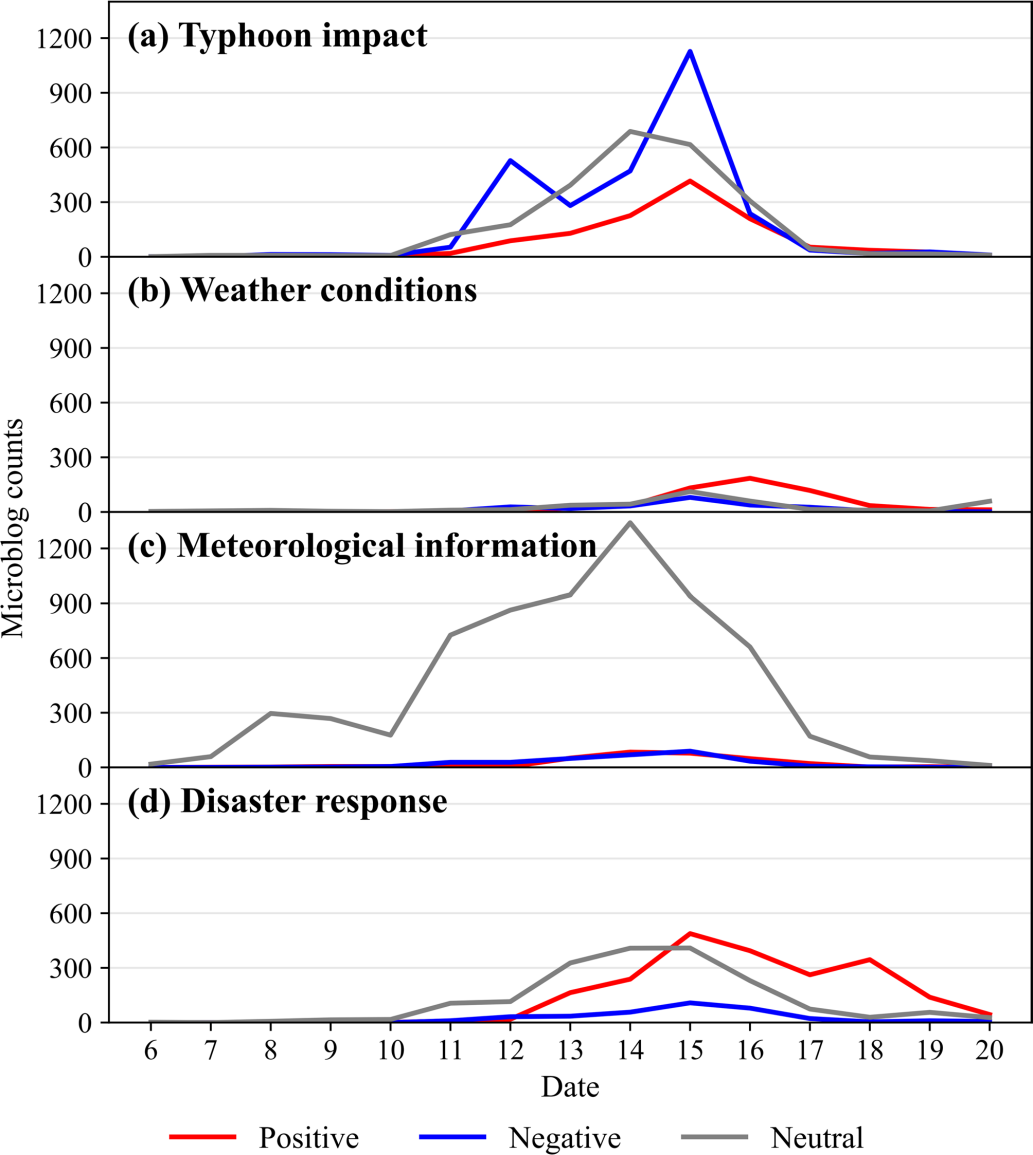
Daily evolution of sentiment within the four topic categories. (**a**) Typhoon impact, (**b**) weather conditions, (**c**) meteorological information, and (**d**) disaster response. Sentiment is denoted as positive (red), negative (blue), and neutral (gray). Statistical analysis from 00:00 on September 6 to 23:59 on September 20, 2022.

The emotional tendencies within the four thematic topics were analyzed by examining both their overall proportion (Fig. 7) and daily evolution (Fig. 8). The key findings are summarized below.

The “typhoon impact” topic exhibited the highest proportion of negative sentiment (43.7%). This aligned with its focus on the disruptions to daily life. Peaks in negative sentiment occurred on September 12 and 15 (Fig. 8a), directly corresponding to periods of peak typhoon intensity and landfall. In contrast, the “weather conditions” topic was dominated by positive sentiment (47.3%). A distinct positive peak emerged after September 16 (Fig. 8b), coinciding with the typhoon’s dissipation and the subsequent sharing of scenery-related posts.

The “meteorological information” topic was overwhelmingly neutral (91.2%). This exceptionally high neutrality underscores the objective and scientific nature of the forecast and warning information disseminated via Sina Weibo. The volume of such information closely tracked the typhoon’s entire lifecycle, demonstrating the timeliness of official meteorological updates. A small initial peak appeared around September 8 as the typhoon formed, followed by a steady increase in posts as warning levels were upgraded. It reached its highest volume concurrent with the issuance of the red typhoon warning on September 14 (Fig. 8c), reflecting the platform’s role in providing authoritative, real-time updates throughout the event.

For the “disaster response” topic, positive (49.0%) and neutral (42.5%) sentiments were predominant, consistent with its focus on reporting relief actions and official updates. A notable increase in positive sentiment began on September 15 (Fig. 8d), aligning with the intensive deployment of rescue efforts and highlighting the platform’s role in sharing constructive news during the response phase.

### Analysis of different account types

User accounts on the Sina Weibo platform were categorized into “official” and “personal” types based on the platform’s public verification system, without applying manual judgment. This system distinguishes accounts primarily through entity attributes. Official accounts are affiliated with identifiable institutions (e.g., government agencies, media outlets, or enterprises). Personal accounts encompass both verified individuals (e.g., celebrities, experts, social media influencers) and unverified general users.

**Fig. 9:**
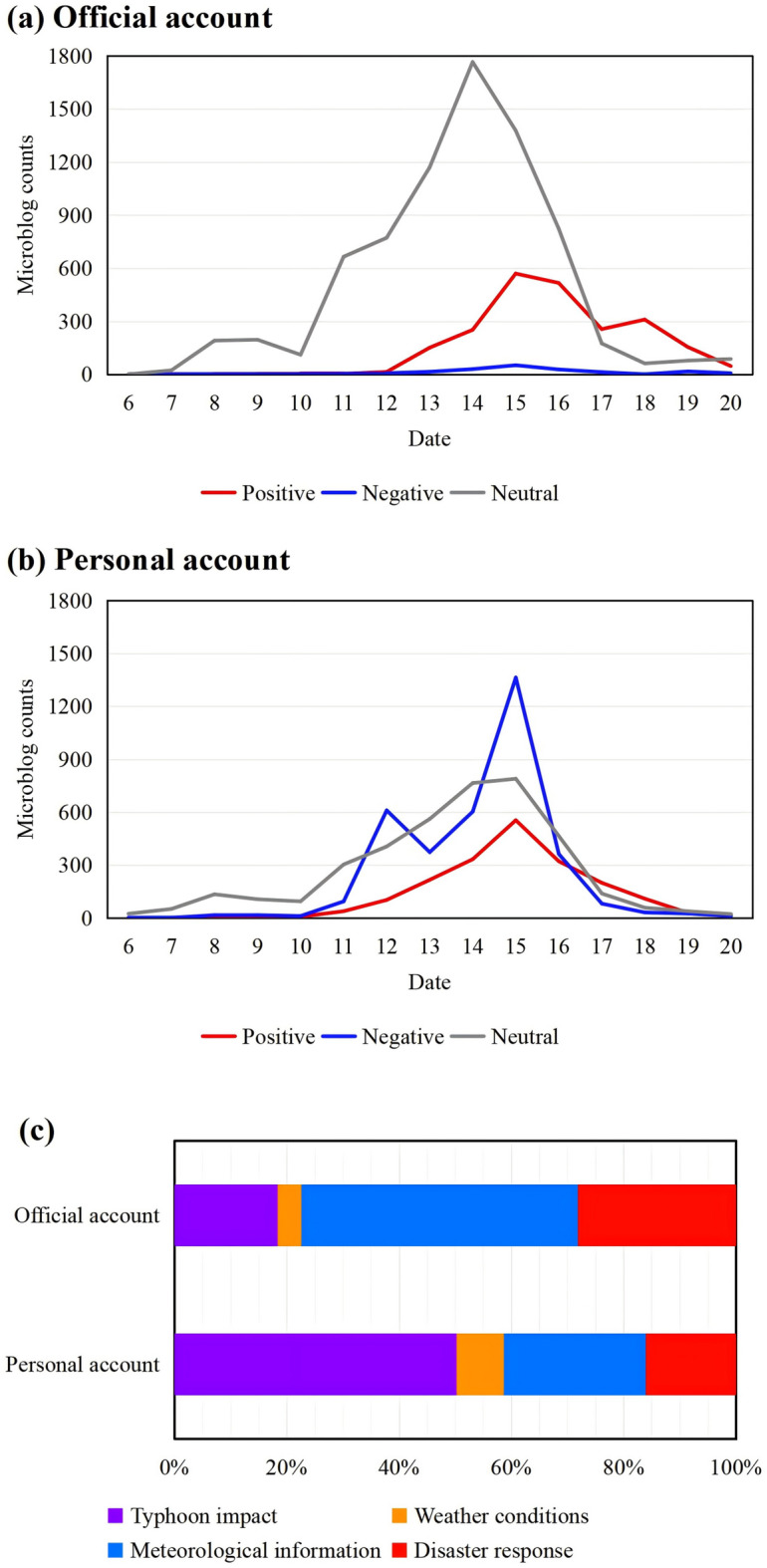
Divergent patterns in content and sentiment between official and personal Sina Weibo accounts. Analysis is based on the platform’s verification system: official accounts (institutional) and personal accounts (individual users, including verified and unverified). (**a**) Sentiment trend for official accounts. (**b**) Sentiment trend for personal accounts. (**c**) Proportional distribution of the four topics across account types. In (**a**) and (**b**), sentiment is denoted as positive (red), negative (blue), and neutral (gray). Statistical analysis from 00:00 on September 6 to 23:59 on September 20, 2022.

Sentiment analysis revealed distinct patterns between these account types. Personal accounts exhibit a more pronounced negative sentiment (Fig. 9a), whereas official accounts demonstrated a significantly higher proportion of neutral content and a lower incidence of negative sentiment (Fig. 9b).

The distribution of topics discussed also differed notably (Fig. 9c). Compared to official accounts, personal accounts contributed a larger share of content related to “typhoon impact” and “weather conditions”. Conversely, official accounts focused more on disseminating information concerning “disaster response” and “meteorological information”. This divergence indicated that information communication during the typhoon event followed distinct pathways: official accounts primarily served to broadcast authoritative warnings and report institutional actions, while personal accounts were more engaged in sharing on-the-ground experiences and personal observations.

### Relationship between Weibo activity and precipitation

This subsection analyzes the relationship between microblog activity and precipitation from September 12 to 18, 2022, across the five provinces/municipalities affected by Typhoon Muifa (Zhejiang, Shanghai, Jiangsu, Shandong, and Liaoning). We calculated Pearson correlation coefficients and their significance (p-values) between daily microblog counts and various precipitation metrics, using the scipy.stats.pearsonr module in Python. To account for multiple comparisons, we applied a False Discovery Rate (FDR) correction^60^ to all 12 correlation tests conducted in this section. Significance is reported based on FDR-adjusted q-values (with q < 0.05 considered significant).

**Fig. 10:**
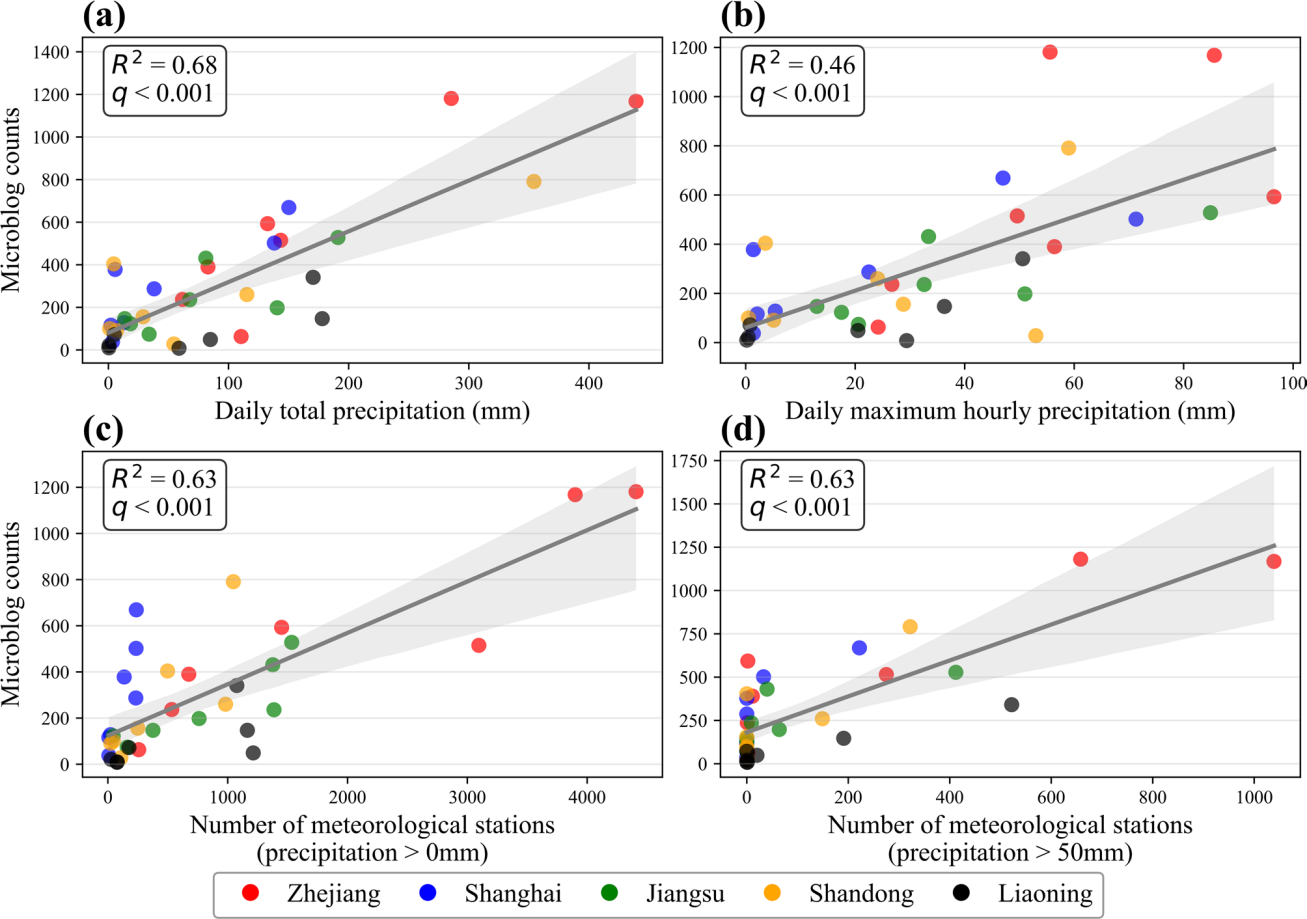
Correlation between daily microblog counts and four precipitation metrics at the provincial level (September 12–18, 2022). (**a**) Daily total precipitation, (**b**) Daily maximum hourly precipitation, (**c**) Number of stations with precipitation > 0 mm, (**d**) Number of stations with precipitation > 50 mm. The grey line indicates the linear fit, and the shaded area is the 95% confidence interval.

Figure 10 shows that daily total precipitation, precipitation coverage (number of stations with > 0 mm precipitation), and precipitation intensity (number of stations with > 50 mm precipitation) all exhibited significant positive correlations with the daily microblog counts (all q < 0.001). The correlation between the daily maximum hourly precipitation was slightly weaker (R^2^ = 0,46; Fig. 10b). Given that daily total precipitation showed the strongest correlation with overall microblog activity in Fig. 10a (highest R^2^, q < 0.001), it was selected as the representative meteorological variable for subsequent analysis.

**Fig. 11:**
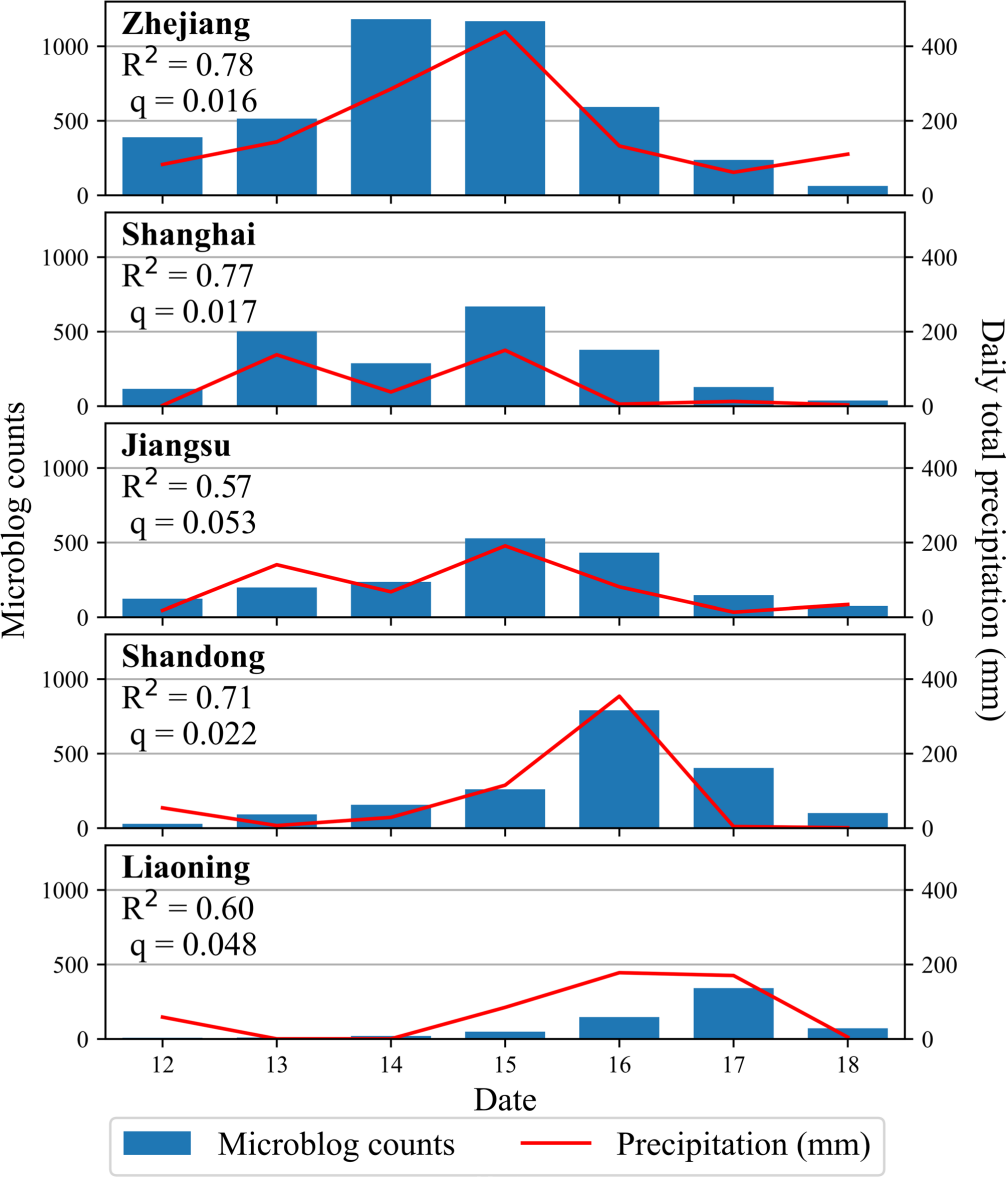
Province-specific correlation between daily total precipitation and daily microblog counts (September 12–18, 2022). Analysis includes the five affected provinces/municipalities: Zhejiang, Shanghai, Jiangsu, Shandong, and Liaoning.

Figure 11 presents the province-specific correlations between daily microblog counts and daily total precipitation. After FDR correction, significant positive correlations were confirmed for the four provinces explicitly forecasted by the China Meteorological Administration as landfall points: Zhejiang (q = 0.016), Shanghai (q = 0.017), Shandong (q = 0.022), and Liaoning (q = 0.048). In contrast, Jiangsu Province, which experienced substantial precipitation but was not listed as a primary landfall point in the official forecasts, showed a statistically non-significant correlation after correction (q = 0.053). This differential pattern suggests that the strength of the association between physical impact (precipitation) and public online response may be moderated by whether a region was highlighted in official risk narratives. The temporal sequence of microblog activity mirrored the typhoon’s northward progression, with earlier-affected provinces showing earlier surges. Furthermore, when precipitation decreased sharply, the decline in microblog posts was more gradual, indicating a “long-tail” effect as public attention persisted beyond the immediate impact.

**Fig. 12:**
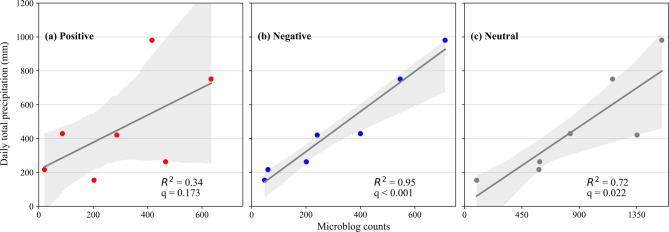
Correlation between daily total precipitation and sentiment-specific microblog counts (September 12–18, 2022). (**a**) Positive, (**b**) Negative, and (**c**) Neutral.

Finally, we correlated precipitation with sentiment orientation. Positive sentiment showed no significant correlation with precipitation (q = 0.173; Fig. 12a). Negative (q < 0.001; Fig. 12b) and neutral (q = 0.023; Fig. 12c) sentiment volumes were significantly correlated with precipitation, with the correlation being stronger for negative content. This suggests that as the typhoon intensified, public discourse shifted toward more negative content. A detailed breakdown revealed that negative sentiment was dominated by the “typhoon impact” topic (1685 out of 2207, 76.3%), while neutral sentiment was primarily associated with “meteorological information” (3319 out of 6160, 53.9%). These findings corroborated the results based on the full dataset (Fig. 7) and indicate that increased discussion of “typhoon impact” drove negative posting, whereas increased posting of “meteorological information” contributed to neutral content.

## Discussion

### Public response to typhoon process on social media

This study illustrates patterns in public risk perception as expressed on social media through a systematic analysis of Sina Weibo content during Typhoon Muifa in 2022. The findings engage with and extend existing theories in three key aspects.

First, the focus and dynamic evolution of public attention correspond to the phased process of the disaster lifecycle and protective action decision-making. Public discussion was highly concentrated on four core topics: typhoon impact, weather conditions, meteorological information, and disaster response. The popularity of these topics shifted in an orderly manner, which aligns with the temporal logic of information diffusion and risk perception rather than occurring randomly. Specifically, discussion under the “meteorological information” topic peaked before the typhoon’s landfall. This peak corresponds precisely to the “pre-decisional information processing” and “core perception formation” stages described by the Protective Action Decision Model (PADM), during which the public actively seeks authoritative forecasts to assess threats^61^. After landfall, the discussion rapidly shifted to “typhoon impact” and “disaster response”, marking the entry into the “decision and behavioral response” stage of the PADM. The sustained activity of the “disaster response” topic after the disaster confirms the extensibility of risk perception. This complete sequence, which progresses from information-seeking to experiential coping and then to a recovery focus, not only validates traditional crisis lifecycle theory but also vividly illustrates how social media functions as a “social sensor” that quantifies disaster evolution processes^62, ^^63^.

Second, a narrative divergence between official and personal accounts co-constructs a dual communication ecology during crises, reflecting the interplay between institutional authority and affective experience. Analysis shows that official accounts dominated the “meteorological information” and “disaster response” topics. Their communication predominantly exhibited a neutral to positive emotional tone, which was designed to convey authoritative information, reduce uncertainty, and build trust in a manner consistent with the principle of constructing “expert power” in crisis communication^61^. In contrast, personal accounts focused on “typhoon impact” and were characterized by a predominantly negative emotional tone that truthfully presented the micro-level experiences and emotions of affected individuals, echoing the social construction perspective of risk perception. This divide is consistent with prior research^63^ and further illustrates that social media serves simultaneously as a top-down channel for strategic risk communication and a bottom-up platform for experiential narratives, together forming a field for meaning construction. Conducting sentiment analysis on these narrative contents can effectively reveal the public’s genuine feelings and evaluations regarding the effectiveness of aid^64^. Therefore, effective emergency communication should not be a one-way dissemination of information but must integrate the rationality of official frameworks with the emotions inherent in personal narratives. Fostering dialogue and complementarity between these two perspectives is essential for achieving collaborative governance that combines rationality and emotion, as well as authority and experience^65^. Risk communication should balance “information sharing” and “emotional resonance”. While the former ensures cognitive consistency, the latter maintains social solidarity and psychological resilience, thereby contributing to a more resilient communication community^66^.

Third, a conditional association exists between physical risk and digital response, and this association is significantly moderated by authoritative risk narratives. This finding deepens our understanding of the complex interactive mechanisms between environmental cues and public interpretation. This study finds an overall positive correlation between precipitation volume and both the volume of social media posts and the prevalence of negative sentiment. From a practical perspective, this correlation highlights the value of integrating meteorological data with social media sentiment for comprehensive monitoring purposes. Such integration enables the near real-time identification of sentiment hotspots and facilitates more spatially and temporally targeted risk communication and emergency resource allocation^62^. Furthermore, provincial-level analysis reveals a critical nuance. In provinces that were explicitly targeted by official forecasts as landing zones, such as Zhejiang and Shanghai, the correlation remained robust and statistically significant even after FDR correction. In contrast, in provinces that also experienced heavy precipitation but were not the focus of forecasts, such as Jiangsu, the statistical significance of the correlation was reduced after FDR correction. This finding strongly challenges the simplified model which posits that physical stimulus directly determines public response, instead supporting a more complex moderating mechanism. It suggests that the “focused risk narrative” constructed by official forecasts acts like a spotlight, which guides and synchronizes public attention and thereby strengthens the observable association between physical impact and digital response^61^. This suggests a potential extension of the PADM model, in which prior authoritative communication may influence how the public interprets subsequent environmental cues.

### Suggestions for improving the efficiency of typhoon meteorological forecast and warning dissemination

Building on the empirical findings regarding the dynamic evolution of public attention, the narrative-emotional divide between official and personal accounts, and the moderating role of authoritative risk narratives in shaping public response, this section proposes targeted recommendations to enhance the efficiency of typhoon forecast and warning dissemination. To address the need for operational specificity, we suggest that emergency management and meteorological agencies consider implementing a time-phased communication strategy aligned with the disaster lifecycle and public information-seeking patterns revealed by our data. Specifically, in the pre-landfall phase (e.g., T-12 h to T-6 h), when public focus is on “meteorological information”, communication should prioritize the clear, frequent, and multi-channel dissemination of authoritative forecasts and preparatory guidance to support public risk assessment. As the threat imminence increases (T-6 h to T-0), messaging must seamlessly integrate concrete protective action directives with empathetic acknowledgment of public concerns, preparing the community for impending impact.

To bridge the identified gap between neutral official updates and negative personal narratives, a critical recommendation is for official accounts to consciously integrate empathy and experiential framing into their core meteorological communications. This involves moving beyond mere fact dissemination to craft messages that acknowledge potential public anxieties, illustrate impacts in relatable terms, and express solidarity with vulnerable groups. For instance, a forecast about heavy rainfall should be coupled with practical advice for affected populations and an expression of concern for outdoor workers, thereby aligning the rational authority of official data with the emotional reality of the public experience.

Concurrently, social media platforms bear significant responsibility for managing the information ecology during such crises. Based on the observed concentration of discussion around key topics, platforms should proactively collaborate with authoritative agencies to feature and amplify official warnings and response updates within relevant topic hubs. Furthermore, to mitigate the risks of misinformation and emotional contagion often associated with personal “impact” narratives, platforms must enhance real-time content moderation, swiftly labeling or removing unverified claims while directing users toward trusted information sources.

Finally, these tactical measures must be underpinned by sustained efforts in public capacity building. The findings underscore the public’s active role as both consumers and amplifiers of risk information. Therefore, long-term public education campaigns led by meteorological departments should focus not only on hazard literacy but also on fostering critical digital media skills. The goal is to cultivate a public that can adeptly navigate the dual communication ecology, rationally processing official guidance while constructively sharing personal experiences to form a more resilient and informed collective response.

## Conclusion and limitations

Analysis of Sina Weibo data during Typhoon Muifa (2022) indicates that the evolution of public opinion on social media aligns with key phases of the typhoon’s lifecycle, with attention surges tied to key forecast updates and landfall events. Topic modeling shows discussion focus shifts orderly from pre-landfall “meteorological information” to “typhoon impact” and “disaster response” afterwards. Negative sentiment correlates strongly with precipitation intensity. Furthermore, personal accounts dominate impact-focused negative narratives, while official accounts maintain neutral tones for forecast dissemination and government action reporting. These findings support the view that social media can serve as a near real-time indicator of public risk perception and behavioral focus during disasters, though further validation across diverse events is needed.

We recommend a time-phased risk communication strategy aligning official messaging with public attention shifts: prioritizing authoritative forecasts pre-landfall, and integrating actionable guidance with empathetic framing as threats intensify. Official channels should bridge narrative gaps by combining factual clarity and public experience acknowledgment; platforms should amplify authoritative content and moderate misinformation. Furthermore, sustained public education is essential to foster digital literacy, enhancing collective resilience and typhoon warning efficiency.

This study is subject to several limitations. First, data collection is constrained by source and format, limited to text-based Sina Weibo content. Future research should include diverse platforms (e.g., WeChat, Douyin) and multimedia analysis (videos, images). Second, data representativeness and accuracy are limited by demographic and geographic factors. The sample is skewed towards younger demographics (over 75% of Weibo users are under 35^67^), potentially excluding older age groups. Spatially, while provincial-level geolocation and normalization using broadband subscriber data help mitigate some regional bias, they mask intra-provincial heterogeneity and limit calibration precision; future studies would benefit from city-level real-time population data for finer-grained analysis. Additionally, some Weibo content originated from non-affected areas, which may introduce noise. Third, keyword/hashtag-based data selection may introduce bias, excluding posts without typhoon-related terms and reducing findings comprehensiveness. Fourth, automated sentiment analysis models lack nuance in interpreting sarcasm, irony, and context-dependent language, risking classification errors. Finally, single-event focus limits generalizability, conclusions may be influenced by Muifa’s track.

Future research should address these limitations by: (1) expanding data sources and incorporating multimedia analysis from diverse platforms; (2) improving sentiment analysis accuracy via advanced models that better capture contextual cues, sarcasm, and irony; (3) enhancing spatial-temporal granularity with precise geolocation data and conducting long-term tracking across multiple disaster events to verify the robustness and generalizability of findings through cross-disaster comparisons; and (4) conducting cross-cultural comparative studies to explore the impacts of socio-cultural and governance factors on disaster-related public opinion dynamics.

## Data Availability

The datasets generated during and/or analysed during the current study are not publicly available due to the constraints of the Sina Weibo Developer Agreement, but are available from the corresponding author on reasonable request.
